# Diagnostic performance of dynamic 3D magnetic resonance angiography in daily practice for the detection of intracranial arteriovenous shunts in patients with non-traumatic intracranial hemorrhage

**DOI:** 10.3389/fneur.2022.1085806

**Published:** 2023-01-27

**Authors:** Arnaud Roumi, Wagih Ben Hassen, Ghazi Hmeydia, Sacha Posener, Johan Pallud, Tarek Sharshar, David Calvet, Jean-Louis Mas, Jean-Claude Baron, Catherine Oppenheim, Olivier Naggara, Guillaume Turc

**Affiliations:** ^1^Neurology Department, GHU Paris Psychiatrie et Neurosciences, Institut de Psychiatrie et Neurosciences de Paris, INSERM U1266, Université Paris Cité, FHU Neurovasc, Paris, France; ^2^Neuroradiology Department, GHU Paris Psychiatrie et Neurosciences, Institut de Psychiatrie et Neurosciences de Paris, INSERM U1266, Université Paris Cité, FHU Neurovasc, Paris, France; ^3^Neurosurgery Department, GHU Paris Psychiatrie et Neurosciences, Institut de Psychiatrie et Neurosciences de Paris, INSERM U1266, Université Paris Cité, FHU Neurovasc, Paris, France; ^4^Neuro-Intensive Care Department, GHU Paris Psychiatrie et Neurosciences, Institut de Psychiatrie et Neurosciences de Paris, INSERM U1266, Université Paris Cité, FHU Neurovasc, Paris, France

**Keywords:** dynamic magnetic resonance angiography, digital subtraction angiography, intracerebral hemorrhage, arteriovenous shunt, vascular malformation, sensitivity, specificity, accuracy

## Abstract

**Introduction:**

Identification of treatable causes of intracranial hemorrhage (ICH) such as intracranial arteriovenous shunt is crucial to prevent recurrence. However, diagnostic approaches vary considerably across centers, partly because of limited knowledge of the diagnostic performance of first-line vascular imaging techniques. We assessed the diagnostic performance of dynamic three-dimensional magnetic resonance angiography (dynamic 3D MRA) in daily practice to detect intracranial arteriovenous shunts in ICH patients against subsequent digital subtraction angiography (DSA) as reference standard.

**Methods:**

We reviewed all adult patients who underwent first-line dynamic 3D MRA and subsequent DSA for non-traumatic ICH between January 2016 and September 2021 in a tertiary center. Sensitivity, specificity, accuracy, positive and negative predictive values of dynamic 3D MRA for the detection of intracranial arteriovenous shunt were calculated with DSA as reference standard.

**Results:**

Among 104 included patients, 29 (27.9%) had a DSA-confirmed arteriovenous shunt [19 pial arteriovenous malformations, 10 dural arteriovenous fistulae; median onset-to-DSA: 17 (IQR: 3–88) days]. The sensitivity and specificity of dynamic 3D MRA [median onset-to-dynamic 3D MRA: 14 (3–101) h] for the detection of intracranial arteriovenous shunt were 66% (95% CI: 48–83) and 91% (95% CI: 84–97), respectively. The corresponding accuracy, positive and negative predictive values were 84% (95% CI: 77–91), 73% (95% CI: 56–90), and 87% (95% CI: 80–95), respectively.

**Conclusion:**

This study suggests that although first-line evaluation with dynamic 3D MRA may be helpful for the detection of intracranial arteriovenous shunts in patients with ICH, additional vascular imaging work-up should not be withheld if dynamic 3D MRA is negative. Comparative prospective studies are needed to determine the best imaging strategy to diagnose arteriovenous shunts after non-traumatic ICH.

## Introduction

Non-traumatic intracranial hemorrhage (ICH) accounts for 28% of overall strokes worldwide (1). It is associated with high mortality rates (up to 40% in the first month) and with high morbidity (only 30% of patients are independent at 6 months) (2, 3). Spontaneous ICH is an heterogeneous entity requiring a thorough etiological work-up. Indeed, although small vessel diseases account for ~80% of non-traumatic ICHs (4), clinicians should not overlook potential focal and treatable underlying causes, particularly arteriovenous shunts such as arteriovenous malformations (AVM) or dural arteriovenous fistulae, which may require endovascular or neurosurgical treatment to prevent recurrence. Indeed, untreated ruptured AVMs are associated with a high risk of recurrence, ~5% per year (5).

Although digital subtraction angiography (DSA) remains the reference standard for the diagnosis of arteriovenous shunts, it is an invasive procedure and thus it is not recommended for all ICH patients. The most recent US Guidelines state that performing DSA is reasonable (a) in patients younger than 70 with lobar spontaneous ICH, (b) in patients younger than 45 with deep or posterior fossa ICH, and (c) in patients aged 45–70 years without history of hypertension and negative non-invasive imaging (moderate recommendation based on non-randomized data) (6).

Various non-invasive imaging modalities based on computed tomography angiography (CTA) or magnetic resonance angiography (MRA) have been proposed for the screening of intracranial arteriovenous shunts (7, 8). In particular, dynamic CTA or MRA have been increasingly used as first-line diagnostic tools to detect shunting vascular malformations (9, 10). However, the diagnostic strategies vary considerably across centers (11), in part because the diagnostic performance of some first-line diagnostic tools -notably dynamic MRA- and the yield of specific diagnostic strategies have been insufficiently studied (12).

The aim of this retrospective study was to assess the diagnostic performance of three-dimensional dynamic MRA (dynamic 3D MRA) compared with subsequent DSA as reference standard for the detection of intracranial arteriovenous shunts in patients with non-traumatic ICH.

## Methods

This article was prepared following the Standards for Reporting of Diagnostic Accuracy Studies (STARD) guidelines (13).

### Population

We reviewed all adult patients who underwent first-line vascular imaging with dynamic 3D MRA and subsequent DSA between January 2016 and September 2021 in a tertiary referral center (GHU Paris Psychiatrie et Neurosciences, Paris, France) as part of the etiological work-up of an acute (< 48 h) non-traumatic ICH. Included patients were hospitalized in the neurology (stroke unit), neurosurgery, or neuro-intensive care departments of the institution, in which MRI is the first-line imaging for suspected stroke. Exclusion criteria were: (1) Isolated subarachnoid, subdural or extradural hemorrhage; (2) isolated intraventricular hemorrhage (because DSA is currently recommended for such patients) (7); (3) concomitant traumatic event; (4) imaging suggestive of hemorrhagic transformation of an ischemic stroke; (5) known intracranial arteriovenous shunt; (6) absolute or relative contraindication to MRI (e.g., MR unsafe implants, clinical instability, severe impairment of consciousness); (7) DSA performed before dynamic 3D MRA, which would have influenced the routine interpretation of the latter; (8) ICH attributed to an intracranial aneurysm.

Medical records were reviewed to collect the following variables: age, sex, hypertension, diabetes mellitus, type of antithrombotic treatment, and clinical severity on admission [National Institutes of Health Stroke Scale (NIHSS) and Glasgow coma scale (GCS) scores], death during hospital stay in our institution. We also reviewed whether a subarachnoid hemorrhage or an intraventricular hemorrhage was associated with the ICH, and if the patient underwent neurosurgery either for hematoma evacuation or external ventricular shunting.

According to French legislation, as this study only implied retrospective analysis of data collected as part of routine care, formal approval by an Ethics Committee was not required.

### Three-dimensional dynamic MRA

The same magnetic resonance (MR) scanner was used throughout the study period [1.5-T superconducting system (Signa Excite Echospeed, GE Healthcare, Waukesha, WI) with 33 mT/m gradient strength], used in conjunction with a multi-channel coil with an 8-channel-receiver radiofrequency system. Three-dimensional dynamic MRA of the affected hemisphere with array spatial sensitivity encoding techniques was performed in the sagittal plane. Twenty dynamic images were obtained to track the contrast bolus. One frame of 20 dynamic images per 1.5 s was repeated 20 times. Imaging parameters for the dynamic images were the following: TR/TE/flip angle, 3/1.2/25°; 300 × 300 mm field of view; 192 × 160 acquisition matrix; slice thickness of 2 mm with 20 overlapped sections resulting in an average 10-cm-thick volume; and a 166.6-kHz bandwidth.

Three-dimensional dynamic MRA was initiated 7 s after the start of a 0.2 mL/kg gadolinium (Dotarem, Guerbet, France) bolus administered intravenously at a rate of 1 mL/s using a Spectris solaris injector (Medrad, Indianola, PA) followed by a 20-mL saline flush. MRA source images were transferred to a workstation (Advantage Windows 4.1, GE Healthcare, Buc, France). Image processing included subtraction of the first volume from the series and reconstruction of the maximum intensity projection. Time from ICH symptoms to dynamic 3D MRA was collected.

### Digital subtraction angiography

DSA was performed in a dedicated biplane neuro-angio suite (General Electrics Innova IGS 630, Buc, France) (14). A 4 or 5-French catheter was used *via* femoral artery approach with a filming rate of 2–4 images per second. Follow-up angiography included a selective injection of internal and common carotid and vertebral arteries in the frontal and sagittal views completed by oblique views when necessary. Images were printed with a 512 × 512 matrix and a field of view of 10–30 cm. For each projection, an 8- to 14-mL bolus of iodinated contrast material (iobitridol, Xenetix, Guerbet, France) was injected at a rate of 5 mL/s using a power injector. Additional 3D acquisition (23-mL, rate of 3 mL/s, rotation speed 40°/s) was performed in selected cases. Time from ICH symptoms to DSA was collected.

### Diagnostic strategy and image analysis

To assess the diagnostic performance of routine dynamic 3D MRA, we decided *a priori* to rely on the original interpretation of dynamic 3D MRA and DSA by a neuroradiologist during daily clinical practice. Arteriovenous shunt on dynamic 3D MRA was defined as visualization of an early venous filling at the arterial phase. The neuroradiologists who interpreted each dynamic 3D MRA were—by definition—blinded to the results of DSA because the latter was performed at a later time point and were not blinded to the other MRI sequences when interpreting dynamic 3D MRA. The neuroradiologists who interpreted each DSA were aware of the results of the dynamic 3D MRA. In selected cases, when the initial etiological explorations (including DSA) were negative, a delayed additional work-up including MRI and DSA was performed at 3 months, or after clot retraction, since mass effect from ICH and edema can obscure small lesions at the acute phase. For those patients who underwent two DSAs, we only considered the result of the second (final) examination as the reference standard.

### Statistical analysis

The distribution of continuous variables was assessed with visual representations (histograms) and the Shapiro–Wilk test. Continuous variables were described as mean ± standard deviation (SD) or median (interquartile range), as appropriate. Categorical variables were expressed as numbers and percentages. DSA was considered the reference standard to detect intracranial arteriovenous shunts. Sensitivity, specificity, accuracy, positive and negative predictive values of dynamic 3D MRA and their 95% confidence intervals (CI) were calculated. Statistical analysis was performed with SAS 9.4 (SAS Institute, Cary, NC).

## Results

A total of 104 patients met our inclusion criteria. Mean age was 51.0 ± 15.0 years, 60 (57.7%) patients were men, and 35 (33.7%) had a history of hypertension (Table 1). On admission, the median NIHSS score was 2 (IQR: 1–7). Eleven (10.6%) patients were treated with antithrombotics at the time of the index event. Sixty-seven (64.4%) patients had a lobar ICH. In 23 (22.1%) and 20 (19.2%) patients, ICH was associated with subarachnoid hemorrhage and/or intraventricular hemorrhage, respectively. Seven (6.7%) patients underwent surgery to evacuate the hematoma (5 patients, 4.9%) or for external ventricular shunt (2 patients, 1.9%). No patient died during their hospital stay in our institution. Median ICH-to-dynamic 3D MRA, MRA-to-DSA, and ICH-to-DSA time delays were 14 (3–101) h, 12 (2–63) days, and 17 (3–88) days, respectively (Table 1). Eight (11.1%) of the 72 patients who had a negative DSA underwent a second DSA, which was negative for each patient. The median time between the first and second DSA was 91 (83–145) days.

**Table 1:** Population characteristics.

**Population characteristics (*n* = 104)**	
Age, mean ± SD, years	51.0 ± 15.0
Male sex	60 (57.7%)
Hypertension	35 (33.7%)
Diabetes mellitus	6 (5.8%)
**Antithrombotic treatment**	
None	93 (89.4%)
Antiplatelet	9 (8.7%)
Anticoagulant	2 (1.9%)
NIHSS score on admission, median (IQR)	2 (1–7)^*^
Glasgow coma score scale on admission, median (IQR)	15 (15–15)^*^
Systolic blood pressure on admission, median (IQR), mmHg	153 (138–171)
Diastolic blood pressure on admission, median (IQR), mmHg	92 (80–104)
ICH onset-to-dynamic 3D MRA time, median (IQR), hours	14 (3–101)
ICH onset to DSA time, median (IQR), days	17 (3–88)
Dynamic 3D MRA to DSA time, median (IQR), days	12 (2–63)
**DSA-confirmed arteriovenous shunt**	
AVM	19 (18.3%)
Dural arteriovenous fistula	10 (9.6%)
**Hematoma location**	
Lobar	67 (64.4%)
Deep	37 (35.6%)
Associated subarachnoid hemorrhage	23 (22.1%)
Associated intraventricular hemorrhage	20 (19.2%)
Neurosurgical Procedure	7 (6.7%)
Death during hospital stay in our institution	0 (0.0%)

Results are expressed as numbers (percentages) except otherwise indicated.

^*^Missing data: NIHSS score on admission (n = 10), GCS on admission (n = 10).

IQR, interquartile range; MRA, magnetic resonance angiography; NIHSS, National Institutes of Health Stroke Scale; SD, standard deviation; DSA, digital subtraction angiography; AVM, arteriovenous malformation.

**Figure 1:**
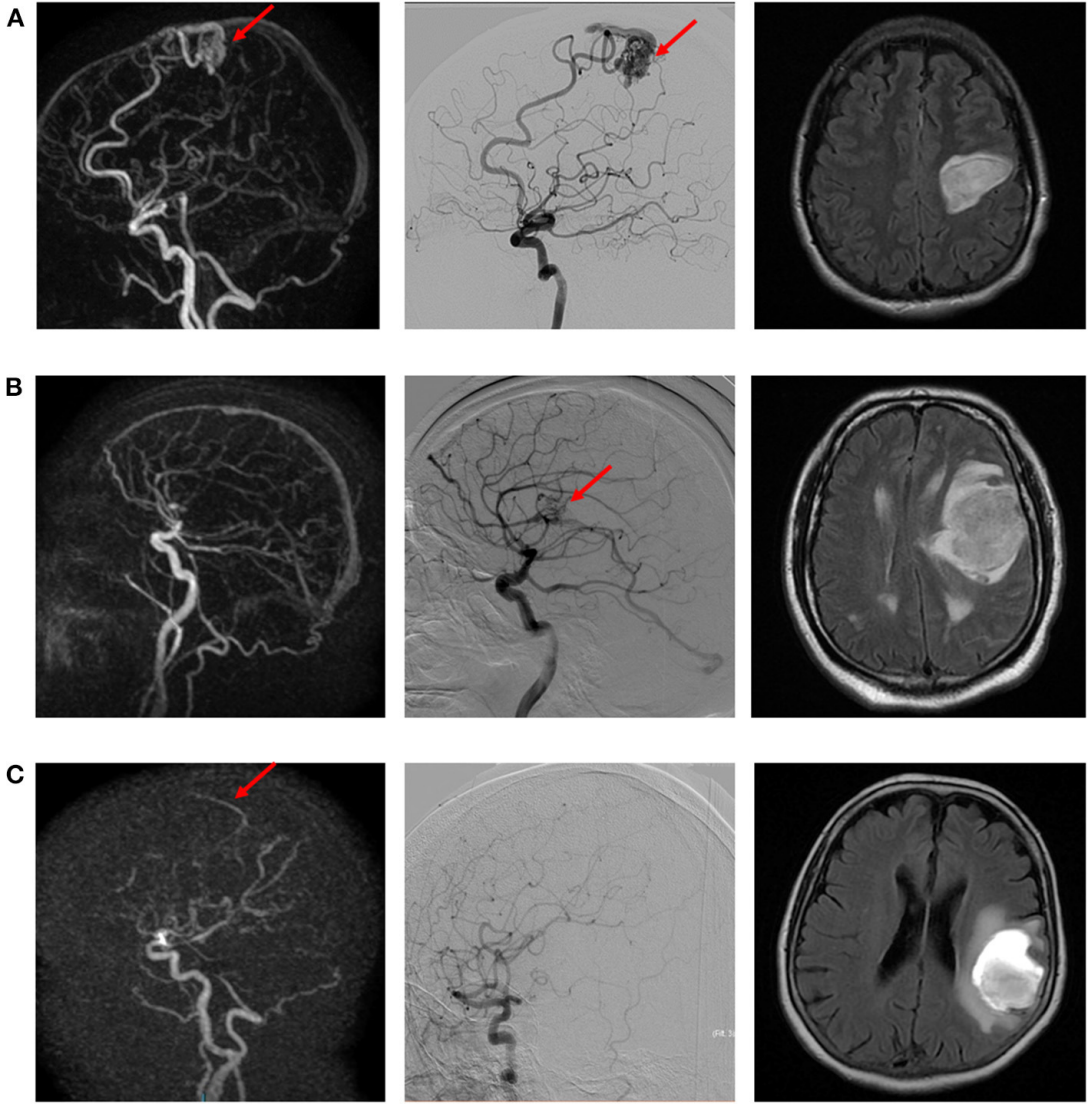
Illustrative examples of true positive **(A)**, false negative **(B)**, and false positive **(C)** cases of intracranial arteriovenous shunt. **(A)** Left precentral AVM suspected on dynamic 3D MRA (left) and confirmed with DSA (middle). Morphological MR (right; FLAIR sequence) depicts the ICH. **(B)** Left opercular AVM diagnosed on DSA (right), not visible on dynamic 3D MRA (left). Morphological MR (right; FLAIR sequence) depicts the ICH. **(C)** Suspicion of early venous enhancement on dynamic 3D MRA (left), not confirmed on DSA. Morphological MR (right; FLAIR sequence) depicts the ICH.

Twenty-six (25.0%) patients had a suspected arteriovenous shunt on dynamic 3D MRA. Twenty-nine (27.9%) patients had a DSA-confirmed arteriovenous shunt, of whom 19 (18.3%) and 10 (9.6%) had a pial arteriovenous malformation and a dural arteriovenous fistula, respectively. Among those 29 patients (Supplementary Table 1), an arteriovenous shunt was suspected on dynamic 3D MRA in 19 (65.5%) cases that were DSA-confirmed (i.e., true positive; Figure 1). Conversely, among the 75 patients with negative DSA, 7 (9.3%) had a dynamic 3D MRA suggesting an arteriovenous shunt, i.e., false positives (Supplementary Table 2). Using DSA as reference standard, the sensitivity and specificity of dynamic 3D MRA for the detection of arteriovenous shunts were 66% (95% CI: 48–83) and 91% (95% CI: 84–97), respectively (Table 2). The corresponding accuracy, positive and negative predictive values were 84% (95% CI: 77–91), 73% (95% CI: 56–90), and 87% (95% CI: 80–95), respectively.

**Table 2:** Diagnostic performance of dynamic 3D MRA for the detection of intracranial arteriovenous shunts in patients with non-traumatic ICH.

**Parameter**	**Value (95% CI)**	**Crude numbers**
Sensitivity	66% (48–83)	19/29
Specificity	91% (84–97)	68/75
Accuracy	84% (77–91)	87/104
Positive predictive value	73% (56–90)	19/26
Negative predictive value	87% (80–95)	68/78

## Discussion

The aim of this retrospective single-center study was to assess the diagnostic performance of dynamic 3D MRA against DSA as reference standard for the early detection of intracranial arteriovenous shunt in the etiological work-up of adult patients with non-traumatic ICH. In this setting, the specificity of dynamic 3D MRA was high (91%) but its sensitivity was modest (66%).

First-line diagnostic strategies for non-traumatic ICH, including the choice of one or multiple imaging techniques, vary considerably across centers. This situation in part reflects that only few studies have compared the diagnostic performance of MR imaging modalities against DSA to detect arteriovenous shunts for acute or recent ICH (see below). Although DSA remains the reference standard, it is invasive and may lead to neurological events in ~1% of patients (6). In our center, the first-line etiological work-up of clinically stable patients with non-traumatic ICH relies on MRI with dynamic 3D MRA, in the absence of contraindication. If it does not suggest an underlying arteriovenous shunt, whether additional DSA should be performed is usually discussed in a multidisciplinary medical meeting. Arguments in favor of performing DSA include the lobar location of ICH, no evidence of small vessel disease in case of deep ICH, no history of hypertension, younger age, and no coagulation impairment.

Data on the diagnostic performance of MRA in spontaneous ICH are limited. In a systematic review, the Cochrane collaboration identified three studies (15–17) comparing MRA and DSA for the detection of vascular abnormalities in general in patients with ICH (18). The reported pooled sensitivity and specificity for detection of vascular malformations were high, namely 98 and 99%, respectively. However, these studies had a relatively modest sample size and included not only patients with arteriovenous shunt but also those with aneurysm or vasculitis. Furthermore, no dynamic imaging was performed in these studies.

Dynamic 3D MRA was first shown to be useful for detecting the appearance of a residual shunt during the follow-up of AVMs treated with either radiosurgery (14) or Onyx embolization (19). It has also proved to be a reliable tool to assess the Spetzler–Martin classification of AVMs and therefore to help determining the best therapeutic strategy in this context (20). Three-dimensional dynamic MRA has been more recently used for the etiological diagnosis of ICH (18), mostly because MRI has become more accessible, is non-invasive, and does not require injection of iodinated contrast agent or exposure to ionizing radiation. However, we could not identify any previous adult study that assessed the diagnostic performance of dynamic 3D MRA compared with DSA for the etiological diagnosis of non-traumatic ICH.

In our study, dynamic 3D MRA had a modest sensitivity for the detection of vascular malformation. This may reflect this imaging modality's modest spatial resolution (21). However, we cannot exclude that the discrepancy between dynamic 3D MRA and DSA may partly reflect the timing applied for each imaging modality. Thus, in our study, DSA was performed later than dynamic 3D MRA (median ICH-to-imaging time of 14 days and 20 h, respectively). This delay could have allowed partial ICH resorption and thus revealed abnormal vessels that were obliterated by the hemorrhage at the time MRA was performed. However, among the 10 patients with a false negative result, only 4 (40%) had a mass effect on MRI.

Nonetheless, our results do suggest that although first-line vascular imaging with dynamic 3D MRA has good accuracy for the diagnosis of arteriovenous shunts, its normality is insufficient to withhold other vascular imaging in patients with non-traumatic ICH. Of note, false positives of dynamic 3D MRA were not uncommon (9.3%), and, although half of them were considered “possible” AVMs (Supplementary Table 1), these results led to performing a DSA. Other non-invasive imaging modalities such as 4D-CTA, which has shown excellent diagnostic accuracy for intracranial arteriovenous shunt compared with DSA (22), may be considered instead of dynamic 3D MRA. We could not identify studies comparing 4D CTA to MRA for the detection of intracranial arteriovenous shunts. Three-tesla dynamic 3D MRA may also provide a better diagnostic yield than 1.5 tesla MRA owing to better spatial and temporal resolution, a point that deserves a specific study. Finally, non-contrast-enhanced 4D MRA with arterial spin labeling seems promising (23), as demonstrated in the characterization of known AVMs (24). Of note, a recent study showed that arterial spin labeling-derived cerebral blood flow maps may have powerful diagnostic value in the etiological workup of pediatric ICH (25).

Our study has limitations. First, our population is not representative of all patients with non-traumatic ICH as shown by the low initial clinical severity (baseline NIHSS score of 2) and the high prevalence of intracranial arteriovenous shunts [28% in our study vs. ~5% in non-selected ICH populations (26)]. It is likely that patients with more severe neurological deficits were not deemed suitable for diagnostic imaging with dynamic 3D MRA by their treating physician, and for instance underwent admission DSA prior to hematoma evacuation, explaining this selection bias. Therefore, whether our findings apply to more severe patients is uncertain. Furthermore, the high prevalence of arteriovenous shunts in our population suggests that in daily practice, dynamic 3D MRA was sometimes considered sufficient to rule out an arteriovenous shunt at our institution (i.e., partial verification bias) (18). This high prevalence may also have artificially increased the positive predictive value of dynamic 3D MRA in our study. However, the high prevalence of arteriovenous shunts is unlikely to have affected sensitivity and specificity, which are generally considered to be independent of the prevalence (27). Second, neither dynamic 3D MRA nor DSA were centrally reviewed. It is possible that an independent review of the images may lead to an improved sensitivity of dynamic 3D MRA. However, the purpose of this pragmatic study was to assess the diagnostic performance of dynamic 3D MRA in routine clinical practice. In this setting, all exams were interpreted by a board-certified neuroradiologist. Third, again matching clinical practice, neuroradiologists who interpreted dynamic 3D MRA were not blinded to others MRI sequences, which could have provided radiological signs for the presence of arteriovenous shunt (e.g., enlarged and dilated serpiginous vessels, direct visualization of the fistulous point/nidus in the vicinity of the hematoma on time-of-flight MRA or post-gadolinium 3D-T1, etc., Supplementary Table 1). In spite of this additional information, the sensitivity of MRI/3D dynamic MRA was modest. Fourth, results of the dynamic 3D MRA were available to the neuroradiologist who interpreted the DSA, which may lead to information bias but again reflects routine practice. Finally, our sample size was modest and therefore the precision of our estimates is limited.

In conclusion, our findings suggest that a first-line evaluation with dynamic 3D MRA may be helpful for the detection of intracranial arteriovenous shunts after non-traumatic ICH, but its normality is insufficient to withhold other vascular imaging work-up. To inform decision-making in the etiological work-up of patients with non-traumatic ICH, prospective studies would be needed to compare the diagnostic performance of non-invasive imaging modalities against DSA as reference standard.

## Data availability statement

The raw, anonymized data that support the findings of this study are available from the corresponding author upon reasonable request, and after signing a data transfer and use agreement. If the data are then used for a publication, the methods should be communicated, and internationally recognized authorship rules should apply.

## Ethics statement

Ethical review and approval was not required for the study on human participants in accordance with the local legislation and institutional requirements. Written informed consent for participation was not required for this study in accordance with the national legislation and the institutional requirements.

## Author contributions

AR: study design, collection and analysis of data, and drafting the manuscript. WB, GH, JP, TS, DC, J-LM, J-CB, CO, and ON: revision of the manuscript for important intellectual content. SP: data collection and revision of the manuscript for important intellectual content. GT: study design, statistical analysis of data, study supervision, and revision of the manuscript for important intellectual content. All authors contributed to the article and approved the submitted version.
