# Supplementary material for: Diagnostic performance of dynamic 3D magnetic resonance angiography in daily practice for the detection of intracranial arteriovenous shunts in patients with non-traumatic intracranial hemorrhage
Source: Front Neurol. 2023 Jan 27;13:1085806. doi: 10.3389/fneur.2022.1085806 (PMC9911434; doi:10.3389/fneur.2022.1085806)
Supplement: Supplementary file 1 [file Table_1.docx]

**Diagnostic performance of dynamic 3D magnetic resonance angiography in daily practice for the detection of intracranial arteriovenous shunts in patients with non-traumatic intracranial hemorrhage**

**Supplementary material**

**Supplementary tables**

**Supplementary Table 1**: Description of the results of dynamic 3D MRA and other MRI sequence of all patients with intracranial arteriovenous shunt confirmed on DSA

| Patient number, sex and age | Type of arteriovenous shunt on DSA | Interpretation of dynamic 3D MRA | Results of other MRI sequences |
| --- | --- | --- | --- |
| Patient #3, M, 43 | Dural arteriovenous fistula | Dural arteriovenous fistula | No abnormalities suggestive of a vascular malformation |
| Patient #4, F, 59 | Dural arteriovenous fistula | Dural arteriovenous fistula | No abnormalities suggestive of a vascular malformation |
| Patient #12, M, 49 | AVM | AVM | Focal enhancement at the periphery of the hematoma (T1 + gadolinium) |
| Patient #22, M, 69 | Dural arteriovenous fistula | Normal | No abnormalities suggestive of a vascular malformation |
| Patient #27, M, 64 | AVM | Normal | No abnormalities suggestive of a vascular malformation |
| Patient #36, F, 50 | AVM | AVM | No abnormality suggestive of a vascular malformation |
| Patient #37, F, 71 | AVM | AVM | Enhancement at the periphery of the hematoma (T1 + gadolinium) |
| Patient #38, F, 36 | AVM | Normal | Enhancement of a small vascular structure in the vicinity of the hematoma (T1 + gadolinium) |
| Patient #40, F, 28 | AVM | AVM | Abnormal vessels on visible on T1 + gadolinium and T2* sequences |
| Patient #43, M, 66 | Dural arteriovenous fistula | Normal | No abnormalities suggestive of a vascular malformation |
| Patient #48, F, 25 | AVM | Normal | No abnormalities suggestive of a vascular malformation |
| Patient #50, M, 19 | AVM | AVM | No abnormalities suggestive of a vascular malformation |
| Patient #52, M, 28 | AVM | AVM | Abnormal vessels visible on T1 (with or without gadolinium injection), T2*, TOF and FLAIR sequences |
| Patient #55, M, 48 | AVM | AVM | No abnormalities suggestive of a vascular malformation |
| Patient #57, F, 50 | AVM | Normal | Abnormal vascular structure visible on TOF and T1 + gadolinium sequences |
| Patient #66, M, 33 | Dural arteriovenous fistula | Dural arteriovenous fistula | No abnormalities suggestive of a vascular malformation |
| Patient #68, M, 24 | AVM | Normal | No abnormalities suggestive of a vascular malformation |
| Patient #73, M, 62 | AVM | Normal | No abnormalities suggestive of a vascular malformation |
| Patient #79, M, 48 | AVM | AVM | Abnormal vessels visible on T1 + gadolinium and T2* sequences |
| Patient #80, M, 23 | Dural arteriovenous fistula | AVM | No abnormalities suggestive of a vascular malformation |
| Patient #86, M, 62 | AVM | Normal | No abnormalities suggestive of a vascular malformation |
| Patient #88, F, 40 | AVM | Normal | No abnormalities suggestive of a vascular malformation |
| Patient #89, M, 33 | Dural arteriovenous fistula | Dural arteriovenous fistula | Abnormal vessels visible on T1 (with or without gadolinium injection), T2*, TOF and FLAIR sequences |
| Patient #92, M, 33 | AVM | AVM | Focal enhancement at the periphery of the hematoma (T1 + gadolinium) |
| Patient #93, M, 67 | Dural arteriovenous fistula | AVM | No abnormalities suggestive of a vascular malformation |
| Patient #100, M, 46 | AVM | AVM | Enhancement of small abnormal vessels (T1 + gadolinium) |
| Patient #101, M, 48 | Dural arteriovenous fistula | Dural arteriovenous fistula | Evidence of a congestion of the deep venous network (hyperintensity of the thalami on FLAIR) + dilatation of deep veins on T1 + gadolinium |
| Patient #102, M, 28 | AVM | AVM | Abnormal vessels visible on T1 (with or without gadolinium injection), T2*, TOF and FLAIR sequences |
| Patient #104, M, 55 | Dural arteriovenous fistula | Dural arteriovenous fistula | No abnormalities suggestive of a vascular malformation |

Abbreviations: AVM: Arteriovenous malformation; DSA: digital subtraction angiography; F: Female; TOF: time of flight; M: Male.

**Supplementary Table 2**: Description of all false positive cases (i.e., intracranial arteriovenous shunt suspected on dynamic 3D MRA but not confirmed on subsequent DSA)

| Patient number, sex and age | Dynamic 3D MRA description |
| --- | --- |
| Patient #2, F, 72 | Wide aspect of the parietal branches of the middle cerebral artery and doubt on an early venous drainage. |
| Patient #42, M, 50 | Possible small arterial nidus facing the posterior and inferior part of the hematoma drained by a relatively dilated nearby cortical vein. |
| Patient #45, F, 26 | Possible mild early enhancement of the torcular |
| Patient #54, M, 66 | Possible arteriovenous micro-shunt detected in the right parieto-occipital sulcus |
| Patient #62, F, 44 | Hypersignal of the left lateral sinus and internal jugular vein, with slightly dilated vascular structures facing the left jugular foramen |
| Patient #90, F, 60 | Early opacification of a left hemispheric cortical vein joining the middle third of the superior sagittal sinus. |
| Patient #95, F, 78 | Possible arteriovenous shunt between the left vertebral artery and a vein communicating with the homolateral lateral sinus. |
